# Fertility preservation in women with cancer: shared decision-making and the essential role of nurses

**DOI:** 10.4069/whn.2025.12.15

**Published:** 2025-12-31

**Authors:** Jeehee Han

**Affiliations:** Red Cross College of Nursing, Chung-Ang University, Seoul, Korea

## Introduction

Advances in medical technology have significantly improved cancer survival rates. As a result, fertility preservation has emerged as an essential component of comprehensive oncology care for patients of reproductive age [1,2]. Fertility extends beyond its biological function and represents a fundamental human desire as well as a core element of personal identity [3]. Consequently, the loss of fertility, or uncertainty regarding future biological parenthood, has a profound impact on identity formation, decision-making, psychosocial well-being, and overall quality of life among cancer survivors [4].

Clinical guidelines recommend counseling regarding fertility risks and preservation options for all reproductive-aged patients prior to initiating gonadotoxic therapy [2]; however, substantial gaps persist in clinical practice. Fertility preservation discussions remain inconsistent, delayed, or omitted altogether, resulting in many patients being unaware of available options or unable to pursue fertility preservation because of time constraints, insufficient information, or inadequate support [5]. Moreover, socioeconomic and geographic inequities continue to limit access to fertility preservation services across diverse populations, underscoring persistent disparities in oncofertility care.

Fertility preservation decision-making requires women to integrate complex medical information with deeply held personal values while simultaneously navigating the emotional distress that accompanies a cancer diagnosis [6]. Within this context, shared decision-making (SDM) represents the most effective framework, as it promotes a collaborative process that synthesizes evidence-based information with value clarification and preference exploration [6,7]. Because SDM depends on continuous communication and supportive engagement, nurses are uniquely positioned to facilitate this process. Their sustained involvement throughout the continuum of care enables them to provide individualized information, emotional support, care coordination, and patient advocacy.

This paper synthesizes current evidence on fertility preservation candidates and strategies, examines these multilevel factors, and proposes an expanded role for nurses in the delivery of patient-centered oncofertility care.

## Clinical and ethical considerations for fertility preservation candidates

Fertility preservation counseling is recommended for individuals of reproductive age at the time of cancer diagnosis and prior to initiating gonadotoxic therapy [2]. Candidates for fertility preservation include those undergoing gonadotoxic chemotherapy; pelvic, abdominal, or total body irradiation; and surgical procedures that may compromise reproductive organs or reproductive function.

Eligibility assessments incorporate patient age, baseline ovarian reserve, treatment urgency, medical contraindications, and reproductive intentions [2]. Importantly, fertility preservation counseling should not be withheld solely on the basis of anticipated survival. Current American Society of Clinical Oncology guidelines recommend fertility preservation counseling for all people with cancer who are at risk of treatment-induced infertility, regardless of prognosis. Withholding this information in the absence of medical contraindications undermines reproductive autonomy, principles of justice, and patient-centered care [2,8,9].

## Fertility preservation methods for women with cancer

The choice of a method is guided by clinical urgency, ovarian reserve, partner status, and patient preferences, with options ranging from established techniques to experimental approaches. For women with cancer, established methods include embryo cryopreservation, oocyte cryopreservation, and ovarian tissue cryopreservation. Embryo cryopreservation yields the highest success rates and is appropriate for individuals with a partner or those willing to use donor sperm [1]. Oocyte cryopreservation offers an effective alternative for individuals without a partner or for those who wish to defer fertilization [10]. Ovarian tissue cryopreservation eliminates the need for ovarian stimulation, accommodates urgent treatment timelines, and can be used in prepubertal girls [11]. Gonadotropin-releasing hormone agonists may provide temporary ovarian suppression during chemotherapy as a potential adjunct to fertility preservation; however, their protective effect remains uncertain, and they should not be considered a substitute for established fertility preservation methods [10].

## Multilevel barriers to shared decision-making in fertility preservation in women with cancer

SDM is the preferred approach for fertility preservation decisions in the context of oncofertility; however, multiple barriers at the individual, professional, and system levels hinder its effective implementation [6,12]. SDM has been shown to reduce decisional conflict and long-term regret while improving satisfaction with cancer-related reproductive decisions [13,14].

### Individual-level barriers

Women with cancer encounter substantial psychological and information-related challenges. Many women have limited health literacy, and emotional distress related to cancer diagnosis and treatment further impairs their ability to process complex oncofertility information [15,16]. Treatment urgency constrains the time available for deliberation and informed decision-making [17]. In addition, high out-of-pocket costs associated with fertility preservation create financial barriers that complicate timely decisions, particularly among women with limited economic resources [6].

### Professional-level barriers

Healthcare providers often have inadequate training and limited knowledge of fertility preservation options for people with cancer, leading to inconsistent counseling practices [18]. Provider biases related to patient age, marital status, socioeconomic status, or prognosis have been associated with selective counseling and unequal access to oncofertility services [19,20]. Moreover, time constraints and absence of structured SDM protocols contribute to substantial variability in oncofertility counseling [21]. Oncology nurses face similar challenges, including knowledge deficits, role ambiguity, and limited confidence in delivering fertility preservation counseling [22].

### System-level barriers

Fragmented care structures and limited multidisciplinary collaboration delay fertility preservation discussions in oncology settings. The absence of designated nurse coordinators or standardized referral pathways further contributes to missed opportunities for people with cancer [23,24]. Financial barriers remain among the most prohibitive obstacles, as fertility preservation procedures are often not fully covered by insurance [25]. Socioeconomic disparities further restrict uptake, with women from lower-income backgrounds experiencing significantly fewer opportunities to access fertility preservation services than those with greater resources [26]. In South Korea, national insurance and infertility support programs subsidize assisted reproductive technologies but largely focus on married couples, leaving the fertility preservation needs of women with cancer inadequately addressed [27]. Although a new government program introduced in 2025 reimburses 50% of the cost of one fertility preservation procedure and provides partial relief, its limited scope means that many women with cancer continue to face substantial out-of-pocket expenses [28].

## Strategies to promote shared decision-making in fertility preservation in women with cancer

Despite these barriers, several facilitators have been identified to enhance the implementation of SDM in fertility preservation, underscoring the need for coordinated strategies across the individual, professional, and system levels.

### Individual-level strategies

Supporting women with cancer begins with the provision of accessible, tailored information regarding fertility preservation options and anticipated outcomes [29]. Women who proactively seek information and ask questions demonstrate higher levels of engagement in SDM discussions. Nondirective communication approaches further support this engagement by encouraging value clarification without steering patients toward specific choices, thereby enabling authentic exploration of personal goals [29,30]. These strategies are most effective when grounded in trust and developed through respectful, transparent, and empathetic provider–patient interactions [17].

### Professional-level strategies

Systematic education and communication training are essential for improving SDM practices. Structured fertility preservation training increases clinicians’ confidence in initiating discussions and improves referral practices [18], while specialized programs enable nurses to implement guidelines effectively and engage patients meaningfully [31]. Beyond content knowledge, training in SDM processes, including eliciting patient values and delivering information with empathy, supports providers in addressing implicit biases and delivering equitable, culturally sensitive counseling across diverse populations.

### System-level strategies

Effective SDM requires integrated infrastructure and sustained institutional commitment. Multidisciplinary teams that include reproductive endocrinologists, mental health professionals, social workers, navigators, and nursing staff are essential for delivering coordinated care [2]. Within these teams, specialized care coordinator nurses bridge oncology and reproductive services by offering decision coaching, psychological support, and navigation assistance [6]. Standardized clinical pathways, supported by electronic health record prompts and mandatory documentation, reduce variability across institutions and help ensure consistency [23]. Furthermore, digital technologies, financial assistance programs, and insurance mandates can expand access and address economic disparities [17]. Continuous quality improvement, informed by patient satisfaction surveys and monitoring of decision regret, enables ongoing refinement of these strategies.

## The essential role of nurses in fertility preservation in cancer care

Based on the evidence reviewed above, I propose that nurses should be recognized as central figures in fertility preservation in cancer care. Nurses maintain continuous interaction with patients throughout the cancer care trajectory, enabling them to fulfill multiple essential roles, including care coordination, patient education, decision support, and advocacy [22]. While oncologists and reproductive specialists typically have brief consultations with patients, nurses maintain ongoing relationships from diagnosis through survivorship. This continuity facilitates the effective implementation of SDM principles. Collectively, these functions address barriers at the individual, professional, and system levels. Nurses are therefore essential to patient-centered fertility preservation care. Their coordinated efforts enhance awareness of fertility preservation options, improve access to services, and support better patient outcomes.

### Care coordination and navigation

Nurses help bridge gaps between fragmented oncology and reproductive medicine systems by coordinating referrals and facilitating timely consultations. Dedicated nurse navigator programs have demonstrated substantial effectiveness, with one institution reporting an eightfold increase in fertility preservation referral rates among cancer patients over a 14-year period [32]. Nurses identify eligible patients, facilitate consultations with reproductive specialists, and coordinate care across disciplines. This systematic approach helps reduce delays in access to fertility preservation services [23].

### Patient education and decision support

Nurses are well positioned to provide tailored education that helps cancer patients understand fertility preservation options and make informed decisions. Through empathetic counseling and nondirective decision coaching, nurses enable women to clarify their values, consider available options, and work through decisional uncertainty without being guided toward specific choices. This personalized support helps patients feel better informed and more confident in their reproductive decision-making.

Evidence supports this approach. Women who receive nurse-led counseling report lower decisional conflict and greater satisfaction with the decision-making process [29]. When nurses facilitate the use of decision aids, cancer patients demonstrate reduced decisional regret as well as enhanced knowledge and self-efficacy related to fertility decisions [13]. These interventions are particularly important because women must process complex medical information while managing the emotional impact of a cancer diagnosis.

### Support for women who choose fertility preservation

For women with cancer who pursue fertility preservation, fertility nurses provide specialized care throughout the treatment process. These nurses guide patients through ovarian stimulation protocols, monitor treatment responses, and address physical and emotional challenges associated with procedures. Fertility nurses coordinate scheduling between oncology and reproductive medicine teams, provide psychosocial support, and manage treatment-related side effects during this time-sensitive process. Nurses also offer ongoing support during survivorship by addressing reproductive concerns that persist after cancer treatment. Research indicates that cancer survivors who receive such support report better outcomes related to fertility-related distress and quality of life [4].

### Patient advocacy and system-level change

In addition to direct patient care, nurses can advocate for improved policies, institutional protocols, and equitable access to fertility preservation resources. The use of specialized care coordinator nurses helps bridge patient-level needs and institutional practices by providing psychosocial support and facilitating SDM processes. By identifying and addressing systemic barriers, such as absent referral protocols or inadequate insurance coverage, nurses contribute to organizational change that benefits patients. Through advocacy efforts, nurses challenge provider biases, identify gaps in care delivery, and participate in quality improvement initiatives. These activities promote standardized referral pathways and more equitable access to fertility preservation services.

## Conclusion

Fertility preservation remains a critical dimension of comprehensive cancer care, extending beyond reproductive biology to encompass patients’ identity, relationships, and future planning. SDM provides an effective framework for integrating fertility preservation into oncology pathways, allowing patients to make informed choices aligned with their values and life goals. However, multilevel barriers across individual, professional, and system domains must be systematically addressed through patient support, clinician education, care coordinator models, and institutional policies that promote equitable access.

Based on the evidence reviewed in this paper, I argue that nurses should be recognized as central figures in fertility preservation care. Their roles as navigators, educators, procedural supporters, and advocates are essential across the oncofertility continuum. Nurses’ sustained involvement throughout care transitions positions them to implement SDM principles in routine practice. Achieving patient-centered oncofertility care therefore requires institutional commitment to integrating trained nurses into formal care pathways, supported by interdisciplinary collaboration and policies that prioritize reproductive autonomy alongside cancer survival.

As survivorship care in oncology continues to advance, recognizing nurses as central figures in fertility preservation is essential for ensuring that all women with cancer are able to make informed decisions about their reproductive future.
